# Safety, efficacy, and mechanism of action of the temporo-masseteric nerve block

**DOI:** 10.22514/jofph.2024.014

**Published:** 2024-06-12

**Authors:** Gayathri Subramanian, Divya Makhija, Sowmya Ananthan, Todd P. Stitik, Samuel Y. P. Quek

**Affiliations:** Department of Diagnostic Sciences, Rutgers School of Dental Medicine, Newark, NJ 07103, USA; Rutgers School of Dental Medicine, Newark, NJ 07103, USA; Center for Temporomandibular Disorders & Orofacial Pain, Department of Diagnostic Sciences, Rutgers School of Dental Medicine, Newark, NJ 07103, USA; Department of Physical Medicine, Rutgers New Jersey Medical School, Newark, NJ 07103, USA

**Keywords:** Temporo-masseteric nerve block, Safety, Efficacy, Masticatory myogenous pain

## Abstract

The objective of the study was to assess the utility and safety of Temporo-masseteric Nerve Block (TMNB), and to explore the mechanism for its apparent sustained pain relief. This manuscript describes, (1) a retrospective study evaluating pain reduction in patients who received the TMNB injection for the management of masticatory myogeneous pain (myalgia, per Diagnostic Criteria for Temporomandibular Disorders (DC/TMD criteria)), and (2) a motor nerve conduction study (NCS) of the temporalis and masseter, performed in the absence of signs or symptoms of TMD, before and after the TMNB injection. The results were as follows. (1) Retrospective study: (n = 186). 52 instances had available baseline and post-TMNB Numerical Pain Rating Scores (NRS) scores, the TMNB injection reduced baseline NRS scores by 70%; pain difference was qualitatively documented in 90 instances (pain relief or improvement in 86/90 instances). 4 instances yielded no pain relief. Mild adverse events recorded included a vasovagal episode (n = 1), transient weakening of blink (n = 2) or burning sensation (n = 1). (2) The Motor NCS demonstrated impairment of the compound-motor-action-potential (CMAP) as recorded from temporalis and masseter muscles following the TMNB injection. In conclusion, the TMNB injection is efficacious and safe. Further studies are warranted to warrant its effectiveness.

## 1. Introduction

Masticatory myalgia or jaw muscle pain commonly involving the temporalis and masseter muscles can arise from local myalgia, myofascial pain or even from more generalized pain disorders like fibromyalgia [1]. Acute masticatory muscle pain is usually secondary to injury, infection or unaccustomed muscle overuse pain and is usually self-limiting [2, 3]. In contrast, the etiopathogenesis of persistent or recurring chronic masticatory muscle pain is more nebulous. The relationship or roles played by several factors, namely, generation of trigger points, parafunctional habit, nociplastic pain, psychosocial burden, sleep alternations, neuroimmune factors and last, but not the least, genetic and epigenetic factors are poorly understood. The biopsychosocial model is a widely accepted model for the etiopathogenesis of chronic myogenous temporomandibular disorders [4]. A variety of empirical treatment strategies are employed, ranging from conservative non-pharmacological interventions such as counseling, occlusal appliances, physical/chiropractic therapy, hypnosis, local interventions such as dry needling, low-level laser therapy, ultrasound, trigger point injections, nerve stimulation, peripheral nerve blocks, acupuncture, to pharmacological interventions including anti-inflammatory medications, antidepressants, skeletal muscle relaxants and benzodiazepines [1, 3]. This manuscript focuses on the Temporo-masseteric Nerve Block (TMNB, previously known as the Twin block), an emerging modality that is not yet validated in the diagnosis and management of myogenous masticatory pain.

The TMNB is a regional local anesthetic nerve block targeting the deep temporal and masseteric branches of V3, the mandibular division of the trigeminal nerve. This regional nerve block technique was first introduced in 2014 and subsequently refined [5, 6]. Emerging evidence supports comparable efficacy of the TMNB injection to trigger point injections in alleviating pain associated with chronic myofascial pain of temporalis and masseteric origin [7, 8].

We report the results from a retrospective review performed to assess the safety and efficacy of pain relief from the TMNB injection administered to patients presenting with acute or chronic myogenous face pain or myalgia of temporalis/masseteric origin [9, 10]. In addition, we present early evidence that the TMNB’s mechanism of action includes, at least in part, motor inhibition of the temporalis and masseter muscles.

## 2. Materials and methods

### 2.1 Retrospective study

IRB approval was obtained to perform the retrospective study (IRB Pro2021000274) of patients presenting to Rutgers School of Dental Medicine/Dental Clinic, The University Hospital, Newark NJ between the years 2014 and 2021, and received the TMNB injection to relieve myogenous pain or myalgia of masseteric or temporalis origin. The data collected included age, sex, acute/chronic pain, baseline and post-TMNB pain levels, and adverse events reported at the time of injection or at a subsequent visit. All instances of administration of the TMNB were reviewed in order to capture any adverse event following its administration. However, based on the completeness of information available for quantitative or qualitative description of baseline and post-TMNB pain, the instances were included for assessing the effect of TMNB on pain alleviation.

### 2.2 Nerve conduction study

A Motor Nerve Conduction Study (NCS) was performed to objectively assess the impact of the TMNB injection on the response to proximal nerve stimulation [11]. A dual-use injectable needle electrode was utilized to deliver an electrical stimulus to the target sitemandible, and the chin respectively to register the compound muscle action of the TMNB injection and, additionally, to deliver 1.8 cc of 2% lidocaine with 1:100,000 epinephrine [12]. Silver nitrate surface electrodes were used as active, reference and ground electrodes over the skin overlying the masseter, potential (CMAP), before and after TMNB administration. A similar configuration was also utilized to record the temporalis muscle as well; in this case, the glabella was the site of the reference electrode.

## 3. Results

### 3.1 Retrospective study

The aim of the retrospective study was to assess the safety and efficacy of the TMNB injection in the setting of acute or chronic masticatory myogenous pain or myalgia of temporalis or masseteric origin. Over 186 instances of TMNB injection were recorded. There were primarily 4 patterns in which the pain response was documented in the electronic health records.

(a) In 52 charts, pain scores as assessed using the Numerical Pain Rating Scale (NRS) were recorded pre- and post-TMNB injection [13]. Three individuals reported mild (NRS 1–3), 10 reported moderate (NRS 4–6), while 39 individuals reported severe baseline pain levels (NRS 7–10). Mean baseline pain was 7.32 +/− 2.07, mean post-Twin block pain was 1.91 +/− 1.85 (mean percentage pain reduction = 72.2 +/− 28%) (Fig. 1). Using a paired *t*-test, a *t*-score of 15.09 with a standard error of difference of 0.36, 51 degrees of freedom and a two-tailed *p* value < 0.0001 was obtained with a mean difference of 5.4904 and a 95% confidence interval ranging between 4.7598 and 6.221.

**Fig. 1.** Pain reduction with the TMNB injection. Mean NRS pain scores at baseline (left) and post-TMNB injection (right) for 52 subjects, with error bars representing standard deviation. NRS: Numerical Pain Rating Scores; TMNB: Temporo-masseteric Nerve Block.

(b) In 11 charts, pain at baseline was documented as NRS scores. Among these, there were 2 reports of mild, 2 of moderate, and 7 of severe baseline pain. Post-TMNB pain levels were qualitatively documented as “pain relieved”, “significantly better” or “pain much more manageable”. Hence, pain reduction could not be quantified objectively in these cases.

(c) In 66 charts, qualitative descriptors of pain were used—the words “extreme”, “excruciating”, “severe”, “throbbing”, “radiating” or “very tender” were interpreted as severe pain (n = 28), “tender”, “persistent”, “sore”, “significant” as moderate (n = 23) and the remainder as mild (n = 15). The words “no pain” or “pain relieved” post-TMNB injection were interpreted to denote effective alleviation, while “pain reduced”, “pain manageable” or “significantly better” were interpreted as incomplete pain relief. The TMNB injection alleviated pain near-totally in 80% of such patients.

(d) Twenty-four charts merely recorded pain relief with TMNB injection while the remainder of the charts did not include pain descriptions.

Notably, in nearly 20% of the patients, a local infiltration of the temporalis tendon insertion along the anterior border of the ramus was necessary to supplement the pain relief from the TMNB injection.

Adverse events: 1 patient experienced a vasovagal reaction, 2 patients experienced a transient weakening of blink (due to palsy of the temporal/zygomatic branch of the facial nerve), 1 patient reported a transient burning sensation at the site of injection and 1 patient reported “heaviness of the eyelid” in the absence of visible signs of any palsy or weakness of the palpebral muscles.

### 3.2 Nerve conduction study

A dental syringe engaging a needle electrode in lieu of a conventional needle was loaded with 1.8 cc of 2% lidocaine with 1:100,000 epinephrine. It was positioned as if to administer the TMNB injection—the needle was introduced through the skin in the desired orientation to depth. A motor nerve conduction study was conducted both right before and after the delivery of the local anesthetic *via* the needle electrode (Fig. 2, right) [14]. Prior to the delivery of the local anesthetic, the participant received an electrical stimulus which elicited the characteristic compound motor action potential (CMAP). The CMAP represents the summated action potential recorded from muscle during a motor nerve conduction study (Fig. 2, left top). After the delivery of the TMNB injection, this CMAP was no longer elicited by a subsequent electrical stimulus (Fig. 2, left bottom). A similar outcome was recorded for the temporalis muscle as well (Fig. 3).

**Fig. 2.** Masseter CMAP before (left top) and after (left bottom) the TMNB injection. (note surface electrode over masseter to record the CMAP) (Figure modified from [2]). TMNB: Temporo-masseteric Nerve Block; CMAP: compound motor action potential.

**Fig. 3.** Temporalis CMAP before (left top) and after (left bottom) the TMNB injection. (note surface electrode over temporalis to record the CMAP) (Figure modified from [2]). TMNB: Temporo-masseteric Nerve Block; CMAP: compound motor action potential.

## 4. Discussion

The TMNB injection has emerged as a novel and practical tool in the diagnosis and management of myalgia or myogenous pain of masseteric and/or temporalis origin [2, 3]. It was originally intended for use primarily as a chairside diagnostic tool to delineate such non-odontogenic pain from odontogenic pain. It was serendipitously observed to provide pain relief that lasted beyond the duration of action of the local anesthetic. Furthermore, a pilot study comparing trigger point injections with the TMNB elicited similar results for sustained pain relief for up to 6 months [7]. This strongly suggested that the TMNB injection had therapeutic potential in the management of myofascial pain of masticatory origin. All these findings underscored the importance of elucidating the mechanism of action of the TMNB local anesthetic injection and ascertaining its safety, especially given its apparent prolonged duration of action.

The retrospective study reported in this manuscript corroborates the safety and acceptability of the TMNB, with minimal and rare adverse effects, supporting its use in routine patient care. In addition, the TMNB administration consistently alleviated pain by nearly 70%, validating its clinical utility. A growing body of evidence suggests that while healthy tendons are devoid of neural fibers, tendons associated with chronic pain demonstrate ingrown nerves and pain neuro-mediators [15]. This is consistent with the occasional need for additional local infiltration around the tendinous muscle insertion for managing chronic myogenous masticatory pain documented in the retrospective study.

It has been the authors’ experience that such infiltration of the temporalis tendon supplements pain relief from the TMNB injection rather than providing the only source of pain relief. It is possible that multiple factors including the nature of the initial pain, chronicity and other unknown factors influence the need for additional pain control from the temporalis tendon infiltration. Rather than arbitrarily exclude such patients from the study, because there is no reliable indication in the chart notes that such injection was the sole reason for the pain relief, the frequency of the need for such an injection to address residual pain was tabulated, as is consistent with our clinical experience.

Rare adverse events from receiving the TMNB injection includes a transient weakening of the blink, that could be explained by proximity to the zygomatic and temporal/frontal branches of the facial nerve, typically avoided with good technique for administering the TMNB injection and knowledge of the regional anatomy [6]. It is notable that these adverse events are mild, transient and rare, and supportive care during the duration of the weakened blink should adequately protect the patient from harm or injury. The duration of such palsy is limited to the duration of the local anesthetic action, that is 60–90 minutes. Supportive therapy such as eye protection by instructing the patient to cover the closed eye with moist gauze and counseling, is adequate. The patient can be supervised in the dental chair until the palsy resolves.

Delivering an electrical stimulus *via* the needle electrode positioned to administer the TMNB injection elicited a compound motor action potential (CMAP) in the target muscles (recorded using the surface electrodes over the temporalis and the masseter). This finding confirms that when the needle is positioned correctly to deliver the TNMB injection, the needle tip is located near the nerve branches innervating both these target muscles, as they traverse the infratemporal fossa in close proximity to one another, before exiting to diverge to innervate their respective targets. Thus, the needle tip delivers the electrical stimulus successfully to each of the target muscles and is able to elicit a CMAP in response. Subsequently, when the local anesthetic is deposited by the same needle electrode in the same location, dampening of the CMAP is recorded on each of the target muscles, providing evidence to the effect of the local anesthetic on the efferent nerves to the masseter and temporalis muscles.

The branches of the mandibular division of V3 innervating the masticatory muscles are mixed nerves, *i.e.*, providing sensory and motor innervation. The nerve conduction study thus performed demonstrates the potential mechanism of action of the TMNB injection to include impediment of motor innervation of its target muscles, the temporalis and the masseter respectively, in addition to blocking nociception from both muscles. However, the duration of this blockade is expected only to be for the duration of the local anesthetic, *i.e.*, approximately 90 minutes for pulpal anesthesia [16]. There are several clinical examples of such reversible motor blockade with administration of the standard dose of local anesthetic, 2% lidocaine with 1:100,000 epinephrine. Documented instances of facial nerve palsy after administering the inferior alveolar nerve block or a masseter trigger point injection typically resolve with termination of the local anesthetic action [17].

We speculate that the protracted pain relief encountered with the TMNB injection, beyond the typical duration of action of the local anesthetic, may stem from a “reset” of the muscular tone of the temporalis and the masseter after the iatrogenic relaxation of the muscle with the local anesthetic injection. Subsequently, if the original cause for the chronic myogenous pain persisted, pain may return, albeit at a lower level, due to some muscle recovery in the interim; we believe that this explanation for pain relief is far more likely than an extended weakening of motor activity itself. Studies further characterizing the TMNB, including surface electromyographic recordings of the masticatory muscles as a time-lapse following a unilateral TMNB injection and measurement of occlusal forces as an outcome of such muscular activity at corresponding time points are ongoing.

Validation of the safety and characterization of the TMNB are essential to promote its acceptance among the dental community as a safe and useful chairside tool to facilitate the diagnosis and management of chronic myofascial/myogenous pain of masticatory origin. In addition, rigorous clinical studies validating its therapeutic potential are warranted. Currently, the diagnosis and treatment of myofascial pain of masticatory origin are complicated and often deferred to oral facial pain specialists [10]. Hence, patients suffer from delayed and restricted access to care, amplifying the burden of such diseases. In contrast, the ability of the TMNB injection to serve as both a diagnostic and therapeutic tool, if validated, will vastly expedite diagnosis, and broaden access to care, because the technique is readily “teachable” to general dental practitioners. Further, the ability to alleviate pain promptly with the TMNB injection, even if temporarily, may complement the care of patients who are being worked up for fabrication of occlusal appliances, for example, by orofacial pain specialists, facilitating pain control until use of the appliance has been optimized.

## 5. Conclusions

In conclusion, this manuscript highlights the potential usefulness of the TMNB injection in alleviating chronic masticatory myalgia or myogenous pain and corroborates its safety and efficacy. In addition, it provides insight into the mechanism of action of the local anesthetic to include interruption of the motor innervation to the temporalis and masseter muscles, in addition to the likely blockade of their sensory innervation as well. These results support the use of TMNB as a diagnostic tool for myogenous masticatory pain or myalgia. Further studies validating its therapeutic efficacy are warranted prior to its incorporation as a therapeutic tool in masticatory myogenous pain.

## 6. Highlights

• The TMNB appears to be efficacious and well-tolerated, with rare, transient adverse effects, in the management of chronic myalgia or myogenous face pain of temporalis/masseteric origin.

• The TMNB’s mechanism of action appears to be, at least in part, impaired motor innervation of the temporalis and masseter muscles.

• If validated, the TMNB can be readily incorporated into clinical practice to facilitate the management of chronic myalgia or myogenous face pain of temporalis/masseteric origin.

## References

[1] Kalladka M, Young A, Khan J. Myofascial pain in temporomandibular disorders: updates on etiopathogenesis and management. Journal of Bodywork and Movement Therapies. 2021; 28: 104–113. 10.1016/j.jbmt.2021.07.015 34776126

[2] Subramanian G, Ananthan S, Zagury JG, Quek SYP. Pragmatic management of myogenous temporomandibular disorder—a narrative review. Journal of Oral and Maxillofacial Anesthesia. 2022; 1: 35.

[3] Zagury JG, Ananthan S, Quek SYP, Subramanian G. Myofascial temporomandibular disorders at a turning point: pragmatic or evidence-based management? Dental Clinics of North America. 2023; 67: 335–348. 10.1016/j.cden.2022.12.003 36965935

[4] Cheatle MD. Biopsychosocial approach to assessing and managing patients with chronic pain. Medical Clinics of North America. 2016; 100: 43–53. 10.1016/j.mcna.2015.08.007 26614718

[5] Quek S, Young A, Subramanian G. The twin block: a simple technique to block both the masseteric and the anterior deep temporal nerves with one anesthetic injection. Oral Surgery, Oral Medicine, Oral Pathology, and Oral Radiology. 2014; 118: e65–e67. 10.1016/j.oooo.2014.01.227 24703404

[6] Quek SYP, Gomes-Zagury J, Subramanian G. Twin block in myogenous orofacial pain: applied anatomy, technique update, and safety. Anesthesia Progress. 2020; 67: 103–106. 10.2344/anpr-67-01-03 PMC7342810 32633773

[7] Ananthan S, Kanti V, Zagury JG, Quek SYP, Benoliel R. The effect of the twin block compared with trigger point injections in patients with masticatory myofascial pain: a pilot study. Oral Surgery, Oral Medicine, Oral Pathology, and Oral Radiology. 2020; 129: 222–228. 10.1016/j.oooo.2019.09.014 32009005

[8] Kanti V, Ananthan S, Subramanian G, Quek SYP. Efficacy of the twin block, a peripheral nerve block for the management of chronic masticatory myofascial pain: a case series. Quintessence International. 2017: 725–729. 10.3290/j.qi.a39094 28990015

[9] Schiffman E, Ohrbach R. Executive summary of the diagnostic criteria for temporomandibular disorders for clinical and research applications. The Journal of the American Dental Association. 2016; 147: 438–445. 10.1016/j.adaj.2016.01.007 PMC4884471 26922248

[10] Schiffman E, Ohrbach R, Truelove E, Look J, Anderson G, Goulet J,* et al*. Diagnostic criteria for temporomandibular disorders (DC/TMD) for clinical and research applications: recommendations of the international RDC/TMD Consortium Network and Orofacial Pain Special Interest Group. Journal of Oral & Facial Pain and Headache. 2014; 28: 6–27. 10.11607/jop.1151 PMC4478082 24482784

[11] Ferrante MA. Neuromuscular electrodiagnosis. Handbook of Clinical Neurology. 2023; 195: 251–270. 10.1016/B978-0-323-98818-6.00019-4 37562871

[12] Subramanian G, Quek SYP, Stitik TP, Ananthan S. Temporo-masseteric nerve block impairs efferent conduction to temporalis and masseter. 2022. Available at: https://iadr.abstractarchives.com/abstract/51am-3667692/temporo-masseteric-nerve-block-impairs-efferent-conduction-to-temporalis-and-masseter (Accessed: 28 April 2024).

[13] Breivik H, Borchgrevink PC, Allen SM, Rosseland LA, Romundstad L, Breivik Hals EK,* et al*. Assessment of pain. British Journal of Anaesthesia. 2008; 101: 17–24. 10.1093/bja/aen103 18487245

[14] Tavee J. Nerve conduction studies: basic concepts. Handbook of Clinical Neurology. 2019; 160: 217–224. 10.1016/B978-0-444-64032-1.00014-X 31277849

[15] Ackermann PW, Alim MA, Pejler G, Peterson M. Tendon pain—what are the mechanisms behind it? Scandinavian Journal of Pain. 2023; 23: 14–24. 10.1515/sjpain-2022-0018 35850720

[16] Becker DE, Reed KL. Local anesthetics: review of pharmacological considerations. Anesthesia Progress. 2012; 59: 90–102. 10.2344/0003-3006-59.2.90 PMC3403589 22822998

[17] Jenyon T, Panthagani J, Green D. Transient facial nerve palsy following dental local anaesthesia. BMJ Case Reports. 2020; 13: e234753. 10.1136/bcr-2020-234753 PMC7478029 32900723

